# Bilateral Ischemic Strokes Secondary to Moyamoya Syndrome Associated With Graves Thyrotoxicosis in a Patient of Amerindian Descent From Peru: A Case Report

**DOI:** 10.7759/cureus.26546

**Published:** 2022-07-04

**Authors:** Jorge Ramírez-Quiñones, Sarah Wahlster, Danny Barrientos-Imán, Ricardo Otiniano-Sifuentes, Pilar Calle-La Rosa, Ana Valencia-Chávez, Carlos Abanto-Argomedo

**Affiliations:** 1 Vascular Neurology Department, Instituto Nacional de Ciencias Neurológicas, Lima, PER; 2 Neurology Department, Anesthesiology and Pain Medicine Department, Neurological Surgery Department, Harborview Medical Center, University of Washington, Seattle, USA

**Keywords:** peru, ischemic stroke, thyrotoxicosis, graves’ disease, moyamoya disease

## Abstract

Moyamoya disease (MMD) is characterized by progressive stenosis of the distal portion of the internal carotid artery and its two main branches, the middle cerebral artery, and the anterior cerebral artery. Clinically, MMD can present with ischemic or hemorrhagic cerebrovascular events. The term Moyamoya syndrome (MMS) is used when the characteristic Moyamoya vasculopathy presents in association with other conditions such as Graves' disease (GD). We report a case of a 34-year-old, right-handed male patient of Amerindian descent. He presented to the emergency room with a two-month history of palpitation, fatigue, and weight loss associated with sudden-onset left hemiparesis, facial asymmetry, and dysarthria. His workup was remarkable for elevated levels of thyroid hormones with the presence of autoantibodies and radiological findings typical of MMS. Moyamoya syndrome in association with Graves' disease has increasingly been noted in Latin American patients and should be considered in the differential diagnosis in the appropriate clinical context.

## Introduction

Moyamoya disease (MMD) is characterized by progressive stenosis of the distal portion of the internal carotid artery (ICA) and its two main branches, the middle cerebral artery (MCA) and anterior cerebral artery (ACA), with the subsequent formation of an abnormal network of collateral arteries [1-2]. The name stems from the characteristic appearance of the abnormally dilated collateral vessels on angiography, similar to a “hazy puff of smoke” (Moyamoya in Japanese) [2-3]. Clinically, MMD can present with ischemic or hemorrhagic cerebrovascular events [1]. The term Moyamoya syndrome (MMS) is used when the characteristic Moyamoya vasculopathy presents in association with other conditions such as cranial radiation exposure, sickle cell disease, trisomy 21, neurofibromatosis-1, or Graves' disease (GD) [1,4]. Most cases of MMS concurring with GD have been reported in Asia, and only three cases from Latin America have been reported so far. Here, we report the first case from Peru, a patient of Amerindian descent who presented with bilateral ischemic strokes, elevated levels of thyroid hormones with the presence of autoantibodies, and radiological findings typical of MMS.

## Case presentation

A 34-year-old, right-handed male patient with no significant past medical history presented to the emergency room with sudden onset weakness in the left hemi-body, facial asymmetry, and dysarthria. The patient had a two-month history of palpitation, fatigue, and weight loss prior to presentation. Physical examination findings included a blood pressure of 135/85mmHg, a heart rate of 95 beats per minute, and a cachectic patient with moist skin and bilateral exophthalmos. His neurological examination was notable for a left hemiparesis and ipsilateral central facial asymmetry, dysarthria, and a positive Babinski's of the left foot, no tremors were noted, and he was conscious, fully alert, and oriented, National Institutes of Health Stroke Scale (NIHSS) score at admission was 5, the modified Rankin Scale (mRS) score was 4 due to the motor deficit.

**Figure 1:**
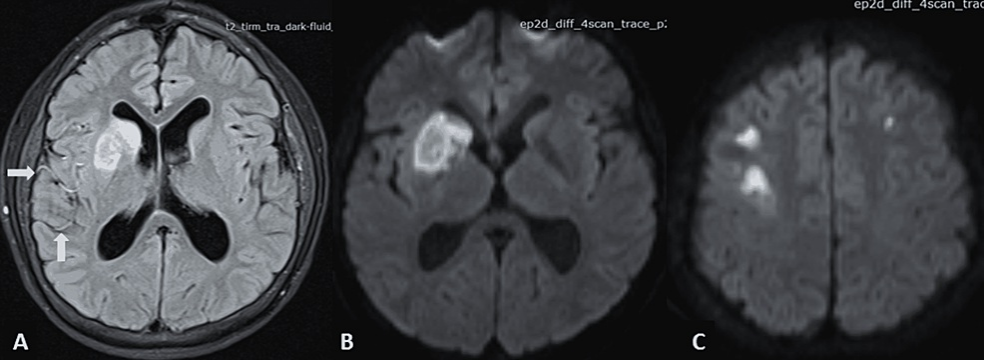
Brain MRI A) FLAIR showing hypersignal in the caudate nucleus, anterior limb of the internal capsule, and right lenticular nucleus, in addition to leptomeningeal enhancement or the “ivy sign” (white arrows). B) DWI showing restricted area. C) DWI with bilateral watershed ischemic areas. FLAIR: fluid-attenuated inversion recovery; DWI: diffusion-weighted imaging

**Figure 2:**
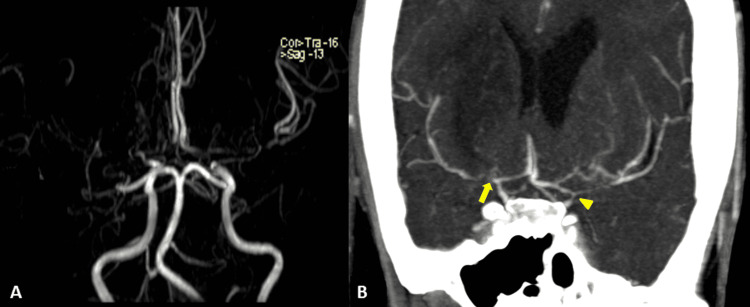
A) TOF MRA showing the absence of flow in the right ICA, MCA, and ACA and decreased flow in the left ICA, MCA, and ACA. B) Angio-tomography with an absence of flow in the right MCA (arrow) and severe stenosis of the left ICA and MCA (arrowhead). TOF MRA: time of flight magnetic resonance angiography; ICA: internal carotid artery; MCA: middle cerebral artery; ACA: anterior cerebral artery

**Figure 3:**
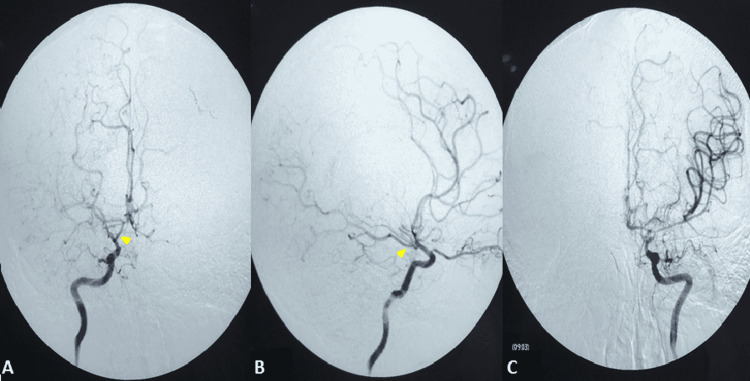
Digital subtraction angiography (A) Right ICA anteroposterior view with supraclinoid ICA stenosis, ACA A1 stenosis, and absence of MCA representation, also visualizing the posterior communicating artery (arrowhead) and posterior cerebral artery filling. (B) ICA lateral view with an absence of MCA representation. (C) Left ICA anteroposterior view with supraclinoid ICA stenosis, ACA A1 segment, and MCA M1 segment stenosis. ICA: internal carotid artery; MCA: middle cerebral artery; ACA: anterior cerebral artery

Lab tests, which include a basic metabolic panel, complete blood count, liver function tests, and urine toxicology were normal, with the exception of the complete thyroid panel (Table 1). Magnetic resonance imaging (MRI) revealed two acute ischemic areas in the anterior circulation (bilateral hemispheres) and bilateral leptomeningeal hyperintensities known as the “ivy sign” (Figure 1A). Time-of-flight (TOF) magnetic resonance angiography (MRA) showed an absence of flow from the right MCA and ACA and decreased flow at the distal portion of the left ICA, ACA, and MCA (Figure 2A). Angio-tomography showed occlusion of the right MCA in its proximal portion and significant stenosis of the ICA and ipsilateral ACA; and significant stenosis in the left ICA, ACA, and MCA (Figure 2B). The digital subtraction angiography in the right carotid axis showed stenosis of the ICA (supraclinoid segment), stenosis of the A1 segment of the ACA, and absence of representation of the MCA (Figures 3A-3B), and in the left carotid axis, it showed stenosis of the ICA (supraclinoid segment), ACA segment A1 stenosis and MCA segment M1 stenosis (Figure 3C). No pathological changes were noted in the vertebrobasilar territory. Transthoracic echocardiography, 24-hour Holter, and carotid duplex studies did not show any abnormalities. The diagnosis was bilateral cerebral infarction due to MMS associated with thyrotoxicosis due to GD. Treatment was started with acetylsalicylic acid 100 mg per day, atorvastatin 80 mg per day, and methimazole 30 mg per day (divided into three doses) while no surgical revascularization was performed. At the three-month follow-up, the patient’s neurological exam had improved with a residual minor hemiparesis; the mRS score was 2.

**Table 1:** Patient's complete thyroid panel and reference values

Parameter	Patient	Reference values
Thyroid-stimulating hormone - TSH	<0.005 IU/ml	0.3-5 IU/ml
Free thyroxine - FT4	6.13 ng/dl	0.9-1.7 ng/dl
Anti-thyroglobulin antibodies	474.8 IU/ml	0-115 IU/ml
Anti-thyroid peroxidase	600 IU/ml	<35 IU/ml
Anti-TSH receptor antibodies	5.72 IU/ml	0-1.75 IU/ml

## Discussion

The term MMS is used to describe the coexistence of the intracranial vascular abnormalities encountered in MMD with other well-documented conditions such as radiation exposure to the head and neck, trisomy 21, neurofibromatosis type 1, sickle cell anemia, and GD [1,4]. It is postulated that there is a causal association between these conditions and the pathogenesis of intracranial vasculopathy. The coexistence of thyroid disease and MMS was first reported in 1983 at the annual meeting of the Japanese Neurology Society, which included three cases with typical radiological findings of MMS and elevated levels of thyroid hormones. The association with GD was first published by Kushima et al.'s study on two young Asian women [5]. Most reports come from Asia, three cases have been reported in Latin America so far (all of them female patients): two in Brazil [6] and one in Colombia [7]. To the best of our knowledge, this is the first report of a Peruvian patient with concomitant MMS and GD. Table 2 summarizes the clinical characteristics, radiological and laboratory findings, and management of the cases reported in South America.

**Table 2:** Clinical characteristics, complementary findings, and management of cases reported in South America with Moyamoya syndrome and Graves' disease CT: computed tomography; ICA: internal carotid artery; MCA: middle cerebral artery; ACA: anterior cerebral artery; NA: not available

	Case 1	Case 2	Case 3	Our case
Sex	Female	Female	Female	Male
Age (years)	22	15	27	34
Previous diagnosis of hyperthyroidism	Yes	No	Yes	No
Clinical presentation	Left hemiparesis, Dysarthria, Headache	Altered mental status, Broca's aphasia, Right hemiparesis	Left hemiparesis, Left-sided hypoesthesia	Left hemiparesis, Dysarthria
Non-contrast head CT	Right MCA territory infarction	Left MCA territory infarction	Right MCA territory infarction	Bilateral infarction of MCA territory
Digital subtraction angiography (DSA)	Left ICA, MCA, and ACA stenosis. Right MCA occlusion.	Left ICA and MCA stenosis	Right ICA, MCA, and ACA stenosis.	Right ICA and ACA stenosis. Right MCA occlusion. Left ICA, ACA, and MCA stenosis.
Thyroid-stimulating hormone - TSH (Ref.:0.3-5 IU/ml)	< 0.03 µUI/mL	< 0.03 µUI/mL	0,05 µUI/mL	<0.005 IU/ml
Thyroxine - T4 (Ref.: 4,5 – 13 µg/dl)	23.4 ug/dl	22.3 ug/dl	21.2 ug/dl	NA
Free thyroxine - FT4 (Ref.: 0.9-1.7 ng/dl)	NA	NA	4.09 ng/dl	6.13 ng/dl
Anti-thyroglobulin antibodies	Positive	Negative	NA	Positive
Anti-thyroid peroxidase	Positive	Positive	NA	Positive
Anti-TSH receptor antibodies	NA	NA	NA	Positive
Hyperthyroidism management	Propylthiouracil, Propranolol	Propylthiouracil	Methimazole, Propranolol, Radioactive iodine	Methimazole
Surgical revascularization	No	No	No	No

The pathophysiological mechanisms that are thought to associate GD with MMS include 1) alteration in the regulation of vascular tone due to increased sensitivity of the sympathetic nervous system [8], and 2) altered T-cell dysfunction; both mechanisms are thought to lead to histological changes of the arterial walls with the progressive appearance of subendothelial fibrosis, intimal thickening and proliferation of smooth cells [9], and hemodynamic changes with alteration of vasoreactivity. Furthermore, it has been reported that GD patients develop vascular changes not only in the typical arteries of the MMS but also more so in the proximal portions of the ICA [10-12], suggesting the existence of a predominantly thyroid vasculopathy in the vessels that receive innervation from the superior cervical ganglion [13]. The reason why these arteries are preferentially affected remains unclear.

Unlike our case, patients with GD and MMS are mostly young women of Asian descent between the ages of 29 and 34 years [8,14-15]. As described by Ohba et al. and Chen et al., ischemic cerebrovascular disease (transient ischaemic attack (TIA) or infarction) is the most frequent form of presentation in this form of MMS [8,14], similar to MMD [16]. From the radiological point of view, cortical and subcortical areas dependent on the anterior circulation of the circle of Willis are affected, predominantly involving the territory of the MCA. Less frequently, the territory of the ACA, as well as the watershed territories, are affected [14-15]. Our patient’s MRI demonstrated bilateral acute ischemic lesions in the anterior circulation, a rare finding only documented on a few occasions in the literature [13,17-18]. The “ivy sign” was also evidenced in the FLAIR sequence, observed as a leptomeningeal hypersignal [19] due to maximum dilation of the pial vasculature to compensate for the decrease in cerebral perfusion pressure in these areas [20]. Angiography showed bilateral vascular damage similar to that of MMD, a finding present in 79% of reported cases [15]. In addition to the neurological manifestations, our case presented with clinical and laboratory manifestations of thyrotoxicosis (reported in up to 45% of cases) and received a de novo diagnosis of Graves' disease, as occurred in 23% of cases in the series by Shah et al. [15].

Therapeutic options include a combination of medical and surgical treatments, including direct or indirect revascularization, and anti-thyroid medication. When revascularization is planned, treatment with thyroid suppressing medications is associated with a better short-term functional prognosis and lower incidence of new ischemic infarcts [14,18]. In addition, some patients are treated with propranolol for the management of autonomic symptoms and tremors. Corticosteroids, as well as other immunosuppressive medications, may also be indicated. The comparison of the clinical results of pharmacological management versus pharmacological-surgical management did not show differences although surgical treatment is recommended in patients with unfavorable clinical and radiological evolution [15]. Our case received only anti-thyroid treatment. Disease progression in MMD versus GD-associated MMS is different. Compared with GD patients, in those with MMS and GD, the progression of the stenosis assessed by angiography is more frequent (40% versus 20.7%) and faster (40 versus 59 months) despite anti-thyroid treatment or surgical treatment [8]. Furthermore, fluctuations in thyroid function or states of thyrotoxicosis are associated with cerebrovascular events due to an increase in brain metabolism and oxygen consumption [8,14-15].

## Conclusions

In conclusion, we report the first case from Peru with a diagnosis of MMS associated with GD in a patient of Amerindian descent. Our case contributes to a growing body of literature describing rare presentations of MMS and may inform future studies about the associations between ethnicity and race with this pathology. MMS represents an infrequent cause of ischemic cerebrovascular disease and, the presence of concomitant diseases, such as GD, should be investigated in the appropriate clinical context to guide management and improve the functional prognosis.

This study was approved by the Instituto Nacional de Ciencias Neurológicas Institutional Review Board. Consent was not required for this case report, including de-identified information appropriately compliant with institutional requirements.
